# Integrated Physiological and Transcriptomic Analyses Responses to Altitude Stress in Oat (*Avena sativa* L.)

**DOI:** 10.3389/fgene.2021.638683

**Published:** 2021-06-17

**Authors:** Yu Jinqiu, Li Bing, Song Tingting, He Jinglei, KongLing Zelai, Lian Lu, He Wenhua, Hai Tao, Huang Xinyu, Liu Zengqing, Cui Guowen, Chen Yajun

**Affiliations:** ^1^Department of Grassland Science, College of Animal Science and Technology, Northeast Agricultural University, Harbin, China; ^2^College of Grassland Science and Technology, China Agricultural University, Beijing, China; ^3^Heilongjiang Academy of Agricultural Sciences, Qiqihar, China

**Keywords:** *Avena sativa* L., altitude stress, transciptome sequence, differentially expressed genes, physiological indexes

## Abstract

Oat is an annual gramineous forage grass with the remarkable ability to survive under various stressful environments. However, understanding the effects of high altitude stresses on oats is poor. Therefore, the physiological and the transcriptomic changes were analyzed at two sites with different altitudes, low (ca. 2,080 m) or high (ca. 2,918 m), respectively. Higher levels of antioxidant enzyme activity, reactive oxygen and major reductions in photosynthesis-related markers were suggested for oats at high altitudes. Furthermore, oat yields were severely suppressed at the high altitude. RNA-seq results showed that 11,639 differentially expressed genes were detected at both the low and the high altitudes in which 5,203 up-regulated and 6,436 down-regulated. Gene Ontology and Kyoto Encyclopedia of Genes and Genomes pathway enrichment tests were conducted and a group of major high altitude-responsive pigment metabolism genes, photosynthesis, hormone signaling, and cutin, suberine and wax biosynthesis were excavated. Using quantitative real-time polymerase chain response, we also confirmed expression levels of 20 DEGs (qRT-PCR). In summary, our study generated genome-wide transcript profile and may be useful for understanding the molecular mechanisms of *Avena sativa* L. in response to high altitude stress. These new findings contribute to our deeper relevant researches on high altitude stresses and further exploring new candidategenes for adapting plateau environment oat molecular breeding.

## Introduction

Extreme environments provide natural laboratories for studies on the processes of speciation and adaptive evolution of organisms (Qiao et al., 2015). The Qinghai-Tibetan Plateau, the highest plateau in the world, plays an important role in climate changes in Asia and even the world (Chi et al., 2019). In particular, the Qinghai-Tibetan Plateau environment is known for its harsh conditions, characterized by severe coldness, intensive ultraviolet radiations, hypoxia, poor soils and low CO_2_ pressure (Xin et al., 2011; Jia et al., 2017). Therefore, the survival in Qinghai-Tibetan Plateau is very challenging for most organisms. Nevertheless, many plant species can thrive in the cold and hypoxic conditions in high-alpine areas (Storz et al., 2010; Yang et al., 2015). Generally, in response to the bioclimatic conditions, plants can make corresponding molecular and physiological changes (Thiebaut et al., 2019). For example, the high-altitudinal gradient can restrict plant growth and reproduction via strong solar UV-B radiation, resulting in a reduction of photosynthetic rates by bleaching chlorophyll a (Chl a) and damaging the photosynthetic apparatus (Zhu and Yang, 2015). As such, some alpine plants are able to activate antioxidants such as APX, CAT, GR, proline and abscisic acid to confer plants with the tolerance to the alpine environments (Ch, 2005; Vinocur and Altman, 2005; Li et al., 2014).

In recent years, transcriptome sequencing has been proven to be an effective and efficient method for determining adaptive evolutions and differential gene expressions in high-altitude plants, e.g., *Kobresia pygmaea, Potentilla saundersiana, Lamiophlomis rotate, and Lobelia.* Some studies demonstrated that alpine plants had various morphological and physiological response strategies to adapt to high-elevation environments. For example, researchers had revealed that some genes were significantly involved in energy metabolism, hypoxia response under positive selection and rapid adaptation (Ma et al., 2015a,b; Zhao et al., 2016). However, up to now, few transcriptome-based investigations have been devoted to the molecular mechanisms of high-altitude adaptation in oats.

Oats have not only high yield, rich nutrition and good palatability, but also the characteristics of cold tolerance, drought resistance and strong adaptability. Oats can germinate when the temperature is 3∼4°C and seedlings can resist the low temperature of −3∼−4°C. Oats are the main forage species in the Qinghai-Tibet Plateau where they are high-quality forages for both winter and spring feeding and disaster-preserving livestock in local farming and pastoral areas. Planting oat grass is of great significance for solving short pasture supply in winter and spring grassland and developing herbivorous livestock and animal husbandry (Sharma et al., 2016). The oat has also been identified as an excellent species that can adapt to various environmental stresses including drought, salinity and pathogen attacks. Oats make morphological and physiological changes to adapt to environments under abiotic stress (Wu et al., 2017; Anwar et al., 2018). However, the adaptation mechanisms of oats in high altitude environments have been rarely studied.

In this study, the first step was designed to characterize the impact of altitudes on oat physiology and agriculture. It showed that high altitude influenced photosynthetic ability, antioxidant enzyme activity, stoma aperture and agricultural parameters of oats. The second step, RNA-seq was employed to study the changes at gene levels between low and high altitudes. According to extensive data analyses, many DEGs and metabolic pathways were identified and characterized which were involved in adaption of oats to high altitude stresses.

## Results

### High Altitude Environments Induced Physiological Changes in Oats

**FIGURE 1:**
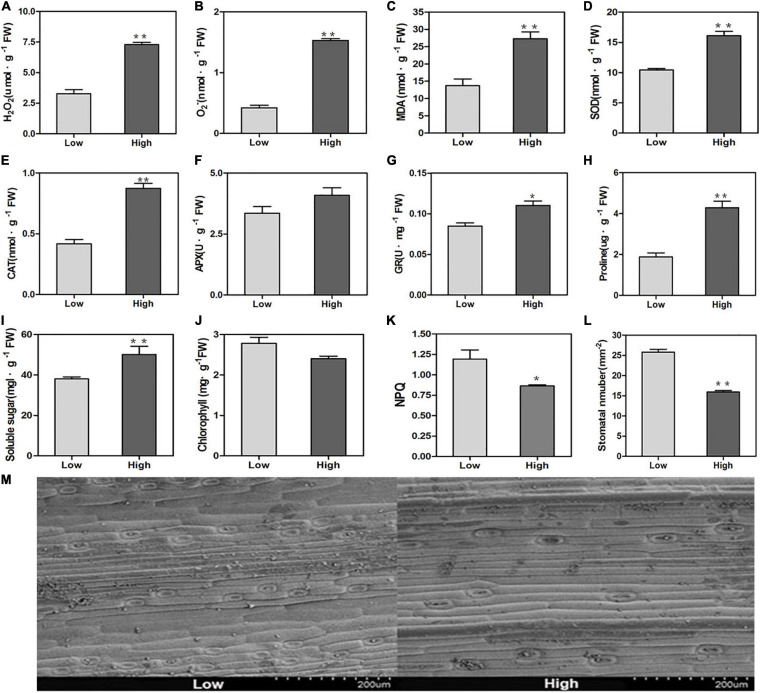
Physiological changes of oats at low and high altitudes. **(A)** H_2_O_2_ activity, **(B)** O_2_^–^ activity, **(C)** MDA activity, **(D)** SOD activity, **(E)** CAT activity, **(F)** APX activity, **(G)** GR activity, **(H)** proline content, **(I)** soluble sugars content, **(J)** chlorophyll content, **(K)** NPQ, **(L)** stomata density. Values represented the means (± SE) from three fully independent biological replicates. Significant differences from the low altitude were denoted by one or two asterisks corresponding to *P* < 0.05 and *P* < 0.01, respectively, by the Student’s *t*-test. **(M)** The photographs of stomata of oats at low and high altitudes.

H_2_O_2_ and O_2_^–^ are essential signals of reactive oxygen species (ROS). They were increased significantly when oats were exposed to high altitude stress (Figures 1A,B). Due to the overproduction of ROS in the cell, MDA is an important marker for lipid peroxidation. The MDA behaviors of high altitude oats were higher than those of low altitude oats (Figure 1C). To further investigate the antioxidant safety mechanism for oats, antioxidant enzyme functions have been investigated. The SOD, CAT, and GR behaviors of the enzymes improved dramatically in high altitude conditions relative to those at low altitude, except for APX (Figures 1D–G). However, the high altitude caused the aggregation of proline osmoregulation substances and soluble sugars in oats (Figures 1H,I).

The chlorophyll content and NPQ at the high altitude decreased and were significantly lower than those at the low altitude (Figures 1J,K). This result indicated that there was a large photosynthetic apparatus damage in high altitude conditions. Stomata control the carbon dioxide absorption and play a crucial role in photosynthesis. This result suggested that the stoma density had significantly decreased at high altitude (Figures 1L,M).

### The Agronomic Traits of Oats Were Remarkably Affected by High Altitude

**FIGURE 2:**
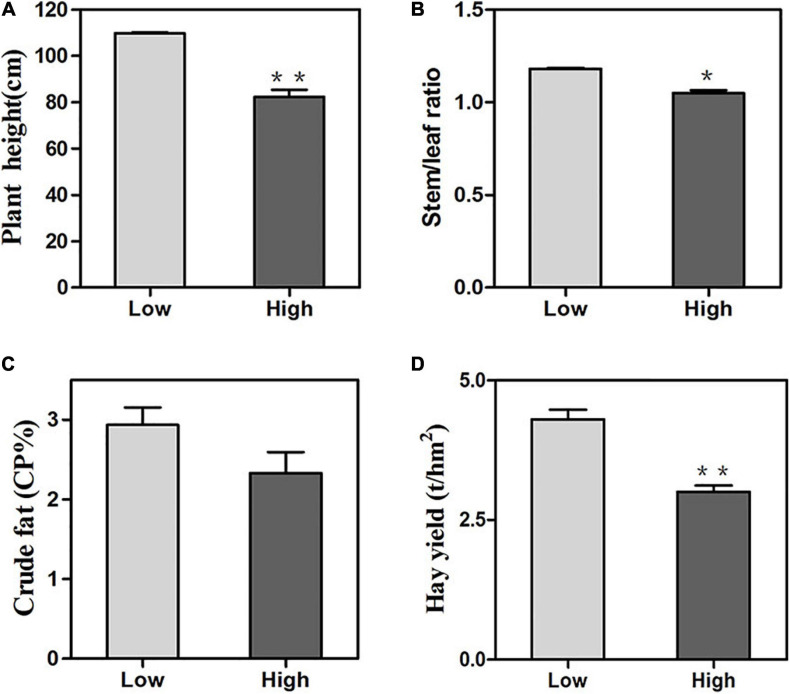
Agronomic trait changes of oats at low and high altitudes. **(A)** plant height, **(B)** crude protein, **(C)** crude fat, **(D)** hay yield. Means were compared using one-way ANOVA. All parameters were shown as the mean ± SE. Significant differences from low altitude were denoted by one or two asterisks corresponding to *P* < 0.05 and *P* < 0.01, respectively by the Student’s *t*-test.

Oats displayed significant differences from two altitudes in terms of plant height, stem/leaf ratio, crude fat and total hay yield. Under high altitude conditions, the plant height of oats was 104.7 cm which was significantly shorter than 126.9 cm at the low altitude (Figure 2A). The stem/leaf ratio and crude fat at the low altitude were 1.18 and 2.7%, respectively, which were significantly different from 1.05 to 1.7% at the high altitude (Figures 2B,C). On the other hand, compared with the low altitude, the high altitude exhibited remarkable decreases in hay yield (Figure 2D). It was clear that oats altered their agronomic traits in two different regions.

### Transcriptome Profiling

To identify the transcriptomes and gene expression profiles of oats at low and high altitudes, six cDNA samples from oat leaves were prepared and sequenced using Illumina HiSeq 2000 platform. A total of 47 50 million raw reads were harvested from each cDNA library. After the low quality reads were removed, 39.49 Gb clean reads were obtained with an average of 6.66 Gb reads for each sample. The percentage of bases higher than Q30 in each sample was not less than 90.91% (Table 1).

**TABLE 1:** Overview of the RNA-sequencing reads generated from each sample.

**Sample**	**Total raw reads (Mb)**	**Total clean reads (Mb)**	**Total clean bases (Gb)**	**Clean reads Q20 (%)**	**Clean reads Q30 (%)**	**Clean READS RATIO (%)**
Low1	50.62	44.36	6.65	96.63	91.03	87.64
Low 2	50.62	44.63	6.69	96.59	90.91	88.16
Low 3	50.61	44.48	6.67	96.41	90.99	87.88
High1	50.62	45.01	6.75	96.77	91.30	88.92
High2	47.92	42.68	6.40	96.86	91.51	89.06
High3	50.62	45.19	6.78	96.78	91.34	89.27

**Low, Low Altitude; High, Higu Altitude; Total Clean Reads (Mb), Filtered reads; Total Clean Bases (Gb), Total number of bases after filtration; Clean Reads Q20 (%), The percentage of the number of bases with mass values greater than 20% in the filtered reads; Clean Reads Q30 (%), The percentage of the number of bases with a mass value greater than 30% in the filtered reads; Clean Reads Ratio (%), The proportion of filtered reads.**

Using Trinity program, these clean reads were then assembled *de novo*, and a cluster deduplication was performed to get the final unigene for a subsequent analysis designated all-unigene. As a consequence, with a mean length of 950, a total of 274,639 All-unigenes were created. Meanwhile, with a GC content of 49.93%, a N50 value of 1,635 bp (Table 2).

**TABLE 2:** Trinity statistics.

**Sample**	**Total number**	**Total length**	**Mean length**	**N50**	**N70**	**N90**	**GC (%)**
Low1	78, 409	58, 299, 780	743	1,206	683	291	50.61
Low 2	73, 260	56, 385, 307	769	1,240	719	304	51.10
Low 3	72, 954	50, 984, 297	698	1,085	626	284	51.25
High1	74, 938	54, 381, 627	724	1,158	656	288	51.29
High2	70, 556	47, 513, 341	673	1,034	590	276	51.53
High3	73, 433	48, 632, 236	662	1,002	576	276	51.01
All-Unigene	274, 639	261, 008, 184	950	1,635	1,062	375	49.93

**Total number, number of reads per sample; Total length, reads length of each sample; Mean length, average length of reads; N50, used to measure the continuity of the assembly, the larger the value, the better the assembly effect; N50, N70, and N90, the percentage of the total length which is the last accumulated value after sorting by the transcript length from big to small and accumulating all transcriptions one by one; GC (%), Ratio of bases G and C.**

**FIGURE 3:**
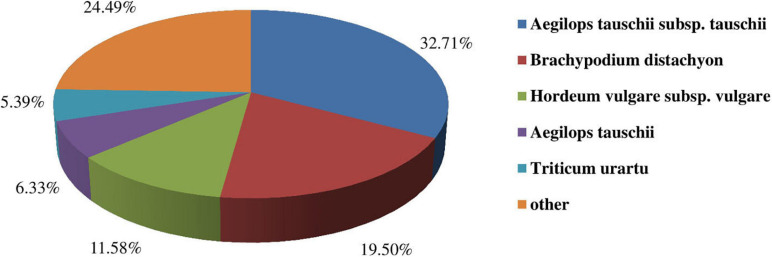
Similarities of Oats to other species.

To predict and analyze the functions of the unigenes, we carried out functional annotations by using BLAST against multiple databases such as Nr, Nt, KEGG, Swissprot, KOG, Interpro, and GO. A total of 94,954 unigenes were successfully matched up in the databases mentioned above. Among them, 11,507 (12.12%), 38,504 (40.55%), 12,044 (12.68%), 11,801 (12.43%), 12,154 (12.80%), 15.199 (16.00%), 49,383 (52.00%) unigenes were found in Nr, Nt, KEGG, Swissprot, KOG, Interpro, and GO databases, respectively (Supplementary Table S1). Moreover, a species distribution map was drawn (Figure 3). The results indicated that oats showed the most similar to *Aegilops tauschii* subsp. *tauschii*, followed by *Brachypodium distachyon* and *Hordeum vulgare* subsp. *vulgare*.

### The Analyses of Differentially Expressed Genes (DEGs)

**FIGURE 4:**
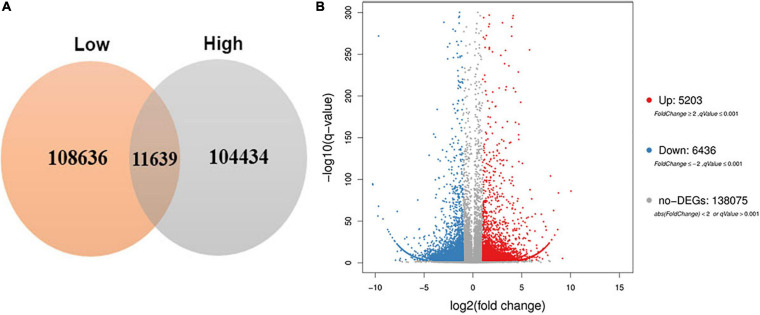
Venn diagram and volcano plot of DEGs at low and high altitudes. **(A)** Venn diagram showed DEGs expressed at each of the two altitude treatments. **(B)** Volcano plot showed the numbers of DEGs identified in comparisons between pairs of libraries.

To obtain the differential expression genes response to high altitude, the Fragments Per Kilobase of transcript, FPKM method was used to analyze the expression abundance of unigenes. As a results, a total of 11,639 differentially expressed genes (|log 2 Fold Change > 2|) were differentially expressed between the low and the high altitudes (Supplementary Table S2 and Figure 4A). Among them, 5,203 DEGs were up-regulated and 6,436 DEGs were down-regulated (Figure 4B). Interestingly, the number of down-regulation genes were more than the up-regulation ones. It indicated that more genes were down-regulated in response to high altitude stresses.

### GO and KEGG Analyses of DEGs

**FIGURE 5:**
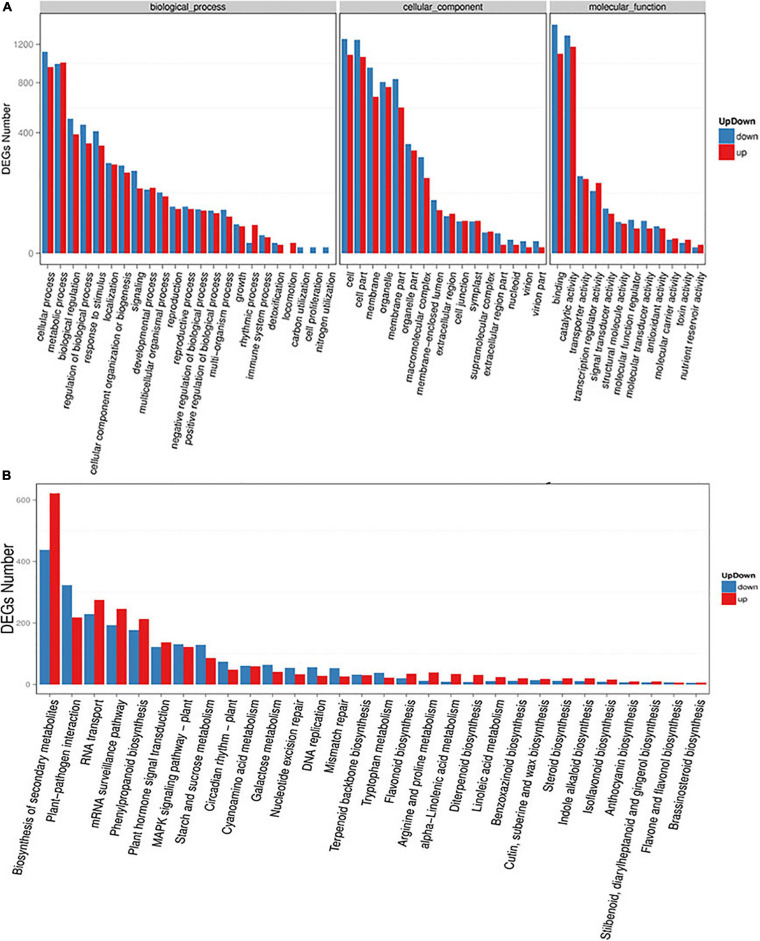
Functional annotation of assembled transcriptome. **(A)** Gene ontology (GO) classifications of the annotated DEGs (up- and down-regulated). **(B)** Kyoto Encyclopedia of Genetics and Genomics (KEGG) database analyses of DEGs (up- and down-regulated) enriched in different biological pathways. The *X*-axis represented enriched pathways and *Y*-axis represented the total number of transcripts.

To investigate the functions of these DEGs under high altitude stresses, we performed GO annotation enrichment analysis using the software package top GO (Supplementary Table S3 and Figure 5A). The results showed that 2,879 transcripts were characterized and annotated with the top GO terms for classified genes being “cellular process” (1,956 transcripts) for biological processes. Other categories with high numbers of genes included “metabolic process” (1,819 transcripts) and “biological regulation” (887 transcripts). These showed that substance synthesis and catabolism, cellular processes, and physiological regulation responses to external stresses were involved in responses of oats to high altitude environmental stresses. In addition, 3,308 transcripts were mapped to interior cell components in which “cell” (2,348 transcripts) and “cell part” (2,314 transcripts) were the most represented categories. The mechanism by which oats responding to high altitude was closely related to the structural composition of their cells, cell membranes and organelles. As for molecular functions, 4,011 transcripts were significantly annotated with the “binding” (2,533 transcripts) and “catalytic activity” (2,478 transcripts) pathways having the most DEGs, respectively. These indicated that the non-covalent bonding of molecules and the catalytic activity of enzymes in biochemical reactions played important roles in the physiological adaptability of oats to high altitude stresses.

At the same time, the DEGs and the biological processes involved in altitude stresses were illuminated and all of the DEGs were queried in KEGG databases. In this study 4,831 transcripts were allocated to 30 pathways in KEGG databases (Supplementary Table S4 and Figure 5B) while “Biosynthesis of secondary metabolites” (1,060 transcripts), “plant-pathogen interaction” (541 transcripts) and “RNA transport” (504 transcripts) pathways were mostly enriched followed by “phenylpropanoid biosynthesis” (390 transcripts), “plant hormone signal transduction” (259 transcripts), “MAPK sigaling pathway” (253 transcripts), and “starch and sucrose metabolism” (215 transcripts). Interestingly most of the genes mapped in the first seven significantly enriched pathways had the trend in down-regulation. These results above indicated that various pathways were involved in responses of oats to different altitudes.

### Analyses of Transcription Factors (TF) in the DEGs

TFs control the expression of numerous genes and play crucial functions in stress-induced signal transduction pathways. In this study, 125 TF genes were differentially expressed between two different altitudes (Supplementary Table S5). Among them, 53 bHLH transcripts, 36 AP2-EREBP transcripts, 11 C_2_C_2_-CO-like transcripts were the top three most TF families. The rest of the transcription factors were ABI3VP1, C_2_C_2_-Dof, C_2_C_2_-GATA, bZIP, C_2_H_2_, ARF, ARR-B, BSD, and C_2_C_2_-YABBY with the numbers of transcripts were 6, 6, 3, 3, 3, 1, 1, 1, and 1, respectively. These results indicated that the TFs above played critical roles in oats response to high altitude stresses.

### Analyses of DEGs Related to Altitudinal Responses

To further identify the key genes related to responses at different altitudes, the DEGs in two pathways for the chlorophyll metabolism and the carotenoid biosynthesis were compared in detail between the low and the high altitudes. The results demonstrated that 23 genes in the porphyrin and the chlorophyll metabolism pathways were detected. Among them, 14 genes had been up-regulated and only 9 genes were down-regulated at high altitude. The DEGs played important roles during the chlorophyll biosynthesis and encoding chlorophyllase (Supplementary Table S6 and Table 3). Moreover, the comparison of the DEGs involved in the carotenoid biosynthesis showed that 32 genes were significantly down-regulated at high altitude (Supplementary Table S7). The results indicated that the photosynthetic pigment biosynthesis was an important process for oats under the high altitude stress.

**TABLE 3:** DEGs associated with pigment metabolism.

**Group**	**Gene ID**	**Regulation**	**Gene description**
	CL3626.Contig4_All	Up	Uroporphyrinogen decarboxylase
	Unigene13229_All	Up	Coproporphyrinogen III oxidase
	CL8939.Contig6_All	Up	Glutamyl-tRNA reductase
	CL9433.Contig7_All	Up	Glutamyl-tRNA synthetase
Chlorophyll and prophyrin metabolism	CL900.Contig8_All	Up	Glucuronosyltransferase
	CL25634.Contig3_All	Up	Glucuronosyltransferase
	CL8418.Contig1_All	Up	Protochlorophyllide reductase
	CL22519.Contig2_All	Up	Chlorophyllase
	CL22519.Contig1_All	Up	Chlorophyllase
	CL5496.Contig2_All	Up	Chlorophyll(ide) b reductase
	CL10250.Contig3_All	Up	Geranylgeranyl diphosphate
	CL10437.Contig1_All	Up	Geranylgeranyl diphosphate
	CL10437.Contig2_All	Up	Geranylgeranyl diphosphate
	CL23991.Contig6_All	Up	Cytochrome c oxidase assembly protein subunit 15
	CL5813.Contig5_All	Down	Porphobilinogen synthase
	CL8939.Contig2_All	Down	Glutamyl-tRNA reductase
	CL9433.Contig14_All	Down	Glutamyl-tRNA synthetase
	Unigene4721_All	Down	Glucuronosyltransferase
	Unigene7840_All	Down	Glucuronosyltransferase
	Unigene16352_All	Down	Glucuronosyltransferase
	Unigene41659_All	Down	Geranylgeranyl-bacteriochlorophyllide a reductase
	Unigene36472_All	Down	Geranylgeranyl-bacteriochlorophyllide a reductase
	CL20435.Contig2_All	Down	Geranylgeranyl-bacteriochlorophyllide a reductase

High altitude stresses might have an impact on plant photosynthesis. In this study, we identified 15 DEGs were significantly regulated in photosynthesis which played a part in photosystem I and II. Meanwhile, 9 genes that mapped to the photosynthesis-antenna protein pathways exhibited differential expressions at high altitude, where the genes encoded light-harvesting complex II chlorophyll a/b binding protein (Supplementary Table S8 and Table 4). These results indicated that changes at the expression levels of the genes above might have blocked photosynthesis, thereby influencing the chlorophyll biosynthesis of oats at high altitude.

**TABLE 4:** DEGs associated with photosynthesis.

**Group**	**Gene ID**	**Regulation**	**Gene description**
PHOTOSYNTHESIS	Unigene34665_All	Up	Photosystem II CP47 chlorophyll apoprotein photosystem II CP47
	Unigene16087_All	Up	Chlorophyll apoprotein
	CL7675.Contig8_All	Up	Photosystem II oxygen-evolving enhancer protein 2
	CL10733.Contig3_All	Up	Photosystem II oxygen-evolving enhancer protein 3
	CL11120.Contig6_All	Up	Photosystem II oxygen-evolving enhancer protein 3
	CL10733.Contig2_All	Up	Photosystem II oxygen-evolving enhancer protein 3
	CL9061.Contig5_All	Up	Photosystem II oxygen-evolving enhancer protein 3
	Unigene24290_All	Up	Photosystem I P700 chlorophyll a apoprotein A1
	CL25957.Contig11_All	Up	Photosystem I P700 chlorophyll a apoprotein A1
	Unigene24271_All	Up	Photosystem I P700 chlorophyll a apoproteinA1/A2
	CL399.Contig3_All	Up	Photosystem I subunit PsaO
	Unigene56697_All	Up	Ferredoxin
	Unigene13484_All	Down	Photosystem II CP47 chlorophyll apoprotein
	Unigene10050_All	Down	Photosystem II CP47 chlorophyll apoprotein
	CL10048.Contig11_All	Down	Ferredoxin–NADP + reductase
Ph Photosynthesis-tenna protein p	Unigene13588_All	Up	Light-harvesting complex II chlorophyll a/b binding protein 1
	Unigene16609_All	Up	Light-harvesting complex II chlorophyll a/b binding protein 1
	Unigene23029_All	Up	Light-harvesting complex II chlorophyll a/b binding protein 1
	Unigene15222_All	Up	Light-harvesting complex II chlorophyll a/b binding protein 1
	CL25362.Contig4_All	Up	Light-harvesting complex II chlorophyll a/b binding protein 1
	Unigene14215_All	Up	Light-harvesting complex II chlorophyll a/b binding protein 1
	Unigene21557_All	Down	Light-harvesting complex II chlorophyll a/b binding protein 1
	Unigene75195_All	Down	Light-harvesting complex II chlorophyll a/b binding protein 5
	CL23904.Contig9_All	Down	Light-harvesting complex II chlorophyll a/b binding protein 7

Hormones are pivotal to plants in stress adaptive signaling cascades and act as central integrators to connect and reprogram different responses. In this experiment, we identified 273 hormone-related DEGs between the low and the high altitude conditions. Among them, 60 genes were identified to encode DELLA protein and acted as a part of gibberellin receptor and phytochrome-interacting factor. Forty-eight genes were identified in the auxin signal transduction pathway in which some encoded auxin-responsive protein IAA and others participated in the process of auxin synthesis and metabolism. In jasmonic acid signal transduction pathway, 41 genes encoded jasmonate ZIM domain-containing protein and transcription factor MYC2. The three pathways above were the most abundant phytohormone signal transduction pathways. In addition, 33 genes in cytokinin pathway, 31 genes in abscisic acid signal pathway, 24 genes in brassinosteroid pathway, 22 genes in ethylene pathway, and 14 genes in salicylic acid pathway were differentially expressed (Supplementary Table S9). The results above suggested that these hormone-related genes can be involved in cell division, stem elongation, stomata aperture, fruit maturation, and disease prevention processes in response to high altitude conditions.

Furthermore, DEGs pathway enrichment study determined that the mechanism of cutin, suberine, and wax biosynthesis was considerably enriched in several genes. Cuticle wax, outside the cell wall of the plant epidermis, is an important hydrophobic structure. Its central aim is to play an important role in plant resistance to abiotic stresses such as salt and heat, but in the cutin, suberine and wax biosynthesis pathways involved in high altitude stress, a total of 32 DEGs were annotated (Table 5). Among them, 2 down-regulated and 1 up-regulated omega-hydroxylase and peroxygenase fatty acid encoding genes were found. We noticed that 10 DEGs of aldehyde decarbonylase and alcohol-forming fatty acyl-CoA reductase were all highly regulated. In addition, 5 (3 up regulated and 2 down regulated) and 14 (4 up regulated and 10 down regulated) omega-hydroxypalmitate O-feruloyl transferase and wax-ester synthase/diacylglycerol O-acyltransferase genes differentially expressed were identified. In addition to high altitude stress in oats, the above findings showed that cutin, suberine and wax biosynthesis played important roles.

**TABLE 5:** DEGs associated with cutin, suberine and wax biosynthesis.

**Group**	**Gene ID**	**Regulation**	**Gene description**
	CL17659.Contig2_All	Up	Omega-hydroxypalmitate O-feruloyl transferase
	CL5973.Contig2_All	Up	Omega-hydroxypalmitate O-feruloyl transferase
	CL5106.Contig4_Al	Up	Omega-hydroxypalmitate O-feruloyl transferase
	CL20119.Contig2_All	Down	Omega-hydroxypalmitate O-feruloyl transferase
	Unigene103114_All	Down	Omega-hydroxypalmitate O-feruloyl transferase
	CL9154.Contig12_All	Up	Peroxygenase
	CL10813.Contig7_All	Down	Fatty acid omega-hydroxylase
	CL10813.Contig5_All	Down	Fatty acid omega-hydroxylase
	Unigene56558_All	Up	Aldehyde decarbonylase
	Unigene56559_All	Up	Aldehyde decarbonylase
	Unigene56562_All	Up	Aldehyde decarbonylase
	CL8485.Contig6_All	Up	Aldehyde decarbonylase
	CL8485.Contig9_All	Up	Aldehyde decarbonylase
	CL8485.Contig2_All	Up	Aldehyde decarbonylase
Cutin,suberine	CL760.Contig4_All	Up	Aldehyde decarbonylase
and wax biosyntesis	CL8485.Contig11_All	Up	Aldehyde decarbonylase
	CL8485.Contig10_All	Up	Aldehyde decarbonylase
	Unigene66294_All	Up	Alcohol-forming fatty acyl-CoA reductase
	Unigene21261_All	Up	Wax-ester synthase/diacylglycerol O-acyltransferase
	CL957.Contig16_All	Up	Wax-ester synthase/diacylglycerol O-acyltransferase
	CL5466.Contig1_All	Up	Wax-ester synthase/diacylglycerol O-acyltransferase
	CL5466.Contig12_All	Up	Wax-ester synthase/diacylglycerol O-acyltransferase
	CL957.Contig29_All	Down	Wax-ester synthase/diacylglycerol O-acyltransferase
	CL5466.Contig5_All	Down	Wax-ester synthase/diacylglycerol O-acyltransferase
	CL957.Contig12_All	Down	Wax-ester synthase/diacylglycerol O-acyltransferase
	CL957.Contig25_All	Down	Wax-ester synthase/diacylglycerol O-acyltransferase
	CL957.Contig21_All	Down	Wax-ester synthase/diacylglycerol O-acyltransferase
	CL957.Contig38_All	Down	Wax-ester synthase/diacylglycerol O-acyltransferase
	CL957.Contig22_All	Down	Wax-ester synthase/diacylglycerol O-acyltransferase
	CL957.Contig40_All	Down	Wax-ester synthase/diacylglycerol O-acyltransferase
	CL957.Contig7_All	Down	Wax-ester synthase/diacylglycerol O-acyltransferase
	CL957.Contig32_All	Down	Wax-ester synthase/diacylglycerol O-acyltransferase

### Relative DEGs Measured by qRT-PCR

**FIGURE 6:**
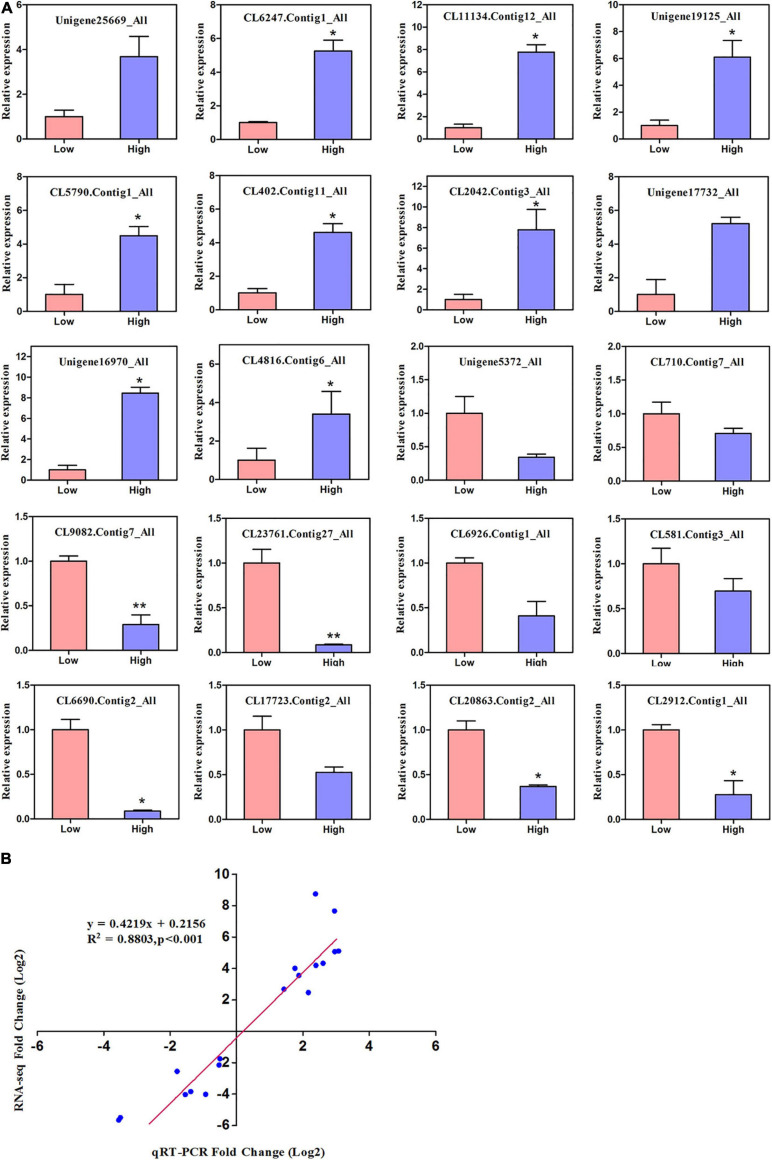
The relative expression levels of 20 DEGs identified in the comparison between RNA-Seq and qRT-PCR. **(A)** The gene relative expression levels were normalized to the expression level of UBC. Error bars represented the standard deviations of three PCR replicates. Significant differences from the low altitude were denoted by one or two asterisks corresponding to *P* < 0.05 and *P* < 0.01, respectively, by the Student’s *t*-test. **(B)** R-values are the correlation coefficients between qRT-PCR and RNA-seq.

We randomly performed qRT-PCR of 20 genes involved in altitude stress to further check the accuracy of RNA-seq data and validate the extent of differential expression genes, as shown in Figure 6A. The transcript abundance of these genes (log 2 fold change) is shown in Supplementary Table S10. Real-time PCR revealed that the expression patterns of 20 low and high altitude genes were compatible with the sequencing findings of expression profiles. A strong correlation (*R*^2^ = 0.88) was observed between the two methods, suggesting that the relevance of RNA-seq data and qRT-PCR showed good accuracy between the two methods (Figure 6B).

## Discussion

The Qinghai-Tibet Plateau itself has unique climate and geographical features which pose huge challenges to the survival of plants. Although plants cannot escape from unfavorable environments, they can change their morphological structures, physiological and molecular processes in which changes happen in endogenous secondary metabolite levels, ion homeostasis, gene expressions and protein activity regulations to adapt to different environmental conditions. Oats are an important source of food and feed in the Qinghai-Tibet Plateau and are beneficial to human health because they are rich in protein and minerals (Noga et al., 2016). Now the data available on the molecular basis of oats response to Qinghai-Tibet Plateau conditions stress is not limited any longer with the development of high-throughput sequencing.

### The Adaptation of the Oat Physiological Characteristics to High Altitude Environments

The increased levels of protective antioxidant enzymes could maintain the homeostasis of ROS. In this study, oats could regulate many physiological processes to adapt to high altitude stresses, e.g., increasing the activities of SOD (Figure 1D) which suggested that oats can improve their ability to resist damages by establishing a better antioxidant enzyme system under high altitude stresses. The membrane lipid peroxidation product MDA was also one of the important indicators of cell damages which could bind to proteins and cause membrane protein denaturation (Yang et al., 2017). Our result showed that oats suffered more severe damages to the cell membrane and antioxidant system in high altitude environments than those at low altitude (Figure 1C). In an alpine environment, low temperatures could affect the osmotic pressure of plant cells. As shown in Figure 1I, high altitude stresses induced the accumulation of soluble sugars in oats. Therefore, soluble sugars might play important roles in oat growth and development and responses to abiotic stresses, and eventually affect yield and quality. In addition, sugars acted as not only an essential source of carbon but also signaling molecules in response to the integration of information from environments as well as developmental and metabolic cues in plants (Lilley et al., 2012; Lastdrager et al., 2014; Ma et al., 2017). Therefore, it could further improve the tolerance of oats under low temperatures at high altitude by increasing the content of soluble sugars to avoid protein coagulation. Meanwhile, oat plants showed sharp decreases in chlorophyll content and NPQ at high altitude (Figures 1J,K). The decreases of chlorophyll content might be because the enhancement of radiation could exacerbate the photooxidation process of photosynthetic pigments or restrain some synthetic processes to decrease the light energy absorbed by leaves at high altitude to avoid or reduce the radiation damages on plants (Lizana et al., 2009). The decrease in NPQ indicated that the plant did not dissipate excess excitation energy in the form of heat, thereby increased the damage on PSII by the accumulation of the excitation energy under high altitude stresses (Costa, 2003). Moreover, the chlorophyll content and NPQ as photosynthesis markers were closely linked to yield which was probably ascribed to the reductions of both plant height and the ratio of stem/leaf, subsequently led to marked decreases in hay yield index (Figure 2). In addition, high altitude conditions could regulate the development of oat plants, e.g., decreased the stomatal density to reduce water loss to adapt to high altitude stress (Mahajan and Tuteja, 2005). This mightbe due to the water shortage caused by low temperatures which was also the result of adaptation to low temperature and strong radiation in high mountains.

### TFs Responding to High Altitude Stresses

When subjected to abiotic, TFs could play an important role by binding to *cis*-regulated DNA elements in gene promoters and activating or inhibiting their expressions (Singh and Laxmi, 2015). bHLH had been reported to directly bond the phytochrome action factor in the photoreceptor signaling network which specifically binded to phytochrome responsive gene promoter G-box regulatory elements *in vitro* (Huq, 2002). In addition, overexpression of *Arabidopsis* bHLH TFs enhanced chilling or osmotic tolerance by reducing malondialdehyde content and accumulating ROS (Liu et al., 2014; Zhao Q. et al., 2018). AP2-EREBP and ABI3 TF could participate in the process of plant growth, development and respond to stresses by regulating the expression of downstream target genes (Liu and Zhang, 2017; Zhao M. et al., 2018). In our study, bHLH, AP2-EREBP, and ABI3VP1 were the three most abundant transcription factors identified as DEGs in response to high altitude stresses. Moreover, many transcription factors containing C_2_C_2_ zinc finger domain were found including C_2_C_2_-CO-like, C_2_C_2_-Dof, C_2_C_2_-GATA, and C_2_C_2_-YABBY. Researches had shown that they played vital biological functions in vegetative tissue development and multiple stress responses (Ge et al., 2011). It could be inferred that bHLH, AP2-EREBP, ABI3VP1, and C_2_C_2_ TFs might play important roles in response to the high altitude stress. In addition, many other TF families were also identified to be differentially expressed such as bZIP, C_2_H_2_, ARF, ARR, and BSD. This also explained that numerous TFs participated actively in the regulation of oats in response to high altitude conditions through different pathways.

### High Altitude Affected the Expression of Genes Related to Pigment Metabolism and Photosynthesis

Plant pigments determine color variations of leaves which are complex biological processes. Among them, chlorophyll and carotenoid are two important antenna pigments in the photosynthetic system. Chlorophyll plays a central role in the process of absorbing and transmitting energy (Tanaka and Tanaka, 2006). Carotenoids can influence chlorophyll content as a major component of the photosynthetic apparatus and photoprotection system. Plateau plants can adapt to UV-B radiation by changing the content of photosynthetic pigments in leaves such as reducing the chlorophyll and carotenoid content (Sicora, 2006). Chlorophyll and carotenoid metabolisms are complex processes involving multiple enzymes. Comparative transcriptome profiling data for the low altitude and the high altitude showed that the expression of numbers of DEGs were significantly regulated including uroporphyrinogen decarboxylase, copropor phyrinogen III oxidase, protochlorophyllide reductase, chlorophyll b reductase, and cytochrome c oxidase assembly protein subunit 15, which were key enzymes associated with the process of chlorophyll synthesis (Mazor et al., 2015). Meanwhile, our comparison of the DEGs involved in carotenoid biosynthesis showed that 32 genes were significantly down-regulated in high altitude (Supplementary Table S3). It might imply that oats can adapt to UV-B radiation stress by changing the content of chlorophyll and carotenoids in leaves.

Chloroplasts are the main sites for photosynthesis which are comprised of membranes, thylakoid system, and stroma with functional units in it. In higher plants, the multi-subunit pigment–protein complexes (i.e., photosystem (PS) I, PSII, light harvesting complexes, cytochrome b6/f, and ATP synthase) are embedded in the highly folded thylakoid membrane where they are responsible for light absorption and energy transfer (Bashir, 2011). Strong radiation in high-altitude environments might have been related to thylakoid development and photosynthesis at the two different altitudes. Based on transcriptome data it was found that genes encoding photosystem II CP47 chlorophyll apoprotein, photosystem II oxygen-evolving enhancer protein, photosystem I P700 chlorophyll a apoprotein and photosystem I subunit PsaO were significantly regulated at high altitude. In higher plants, light-harvesting complex II chlorophyll a/b binding proteins that accumulate in the thylakoid membranes, where their proposed function is in photoprotection (Hutin, 2003; Beck et al., 2017; Ozawa et al., 2018). The chlorophyll a/b-binding proteins are correlated with photodamage in the PSII reaction centers (Heddad et al., 2006). We found that the proteins were significantly enriched suggesting that strong photosynthetic apparatus damage occur in high altitude (Table 3). These results were consistent with our previous photosynthetic physiological index analysis (Figure 2B) which further demonstrated that the genes related to pigment metabolism and photosynthesis in oats could respond to high altitude stresses.

### Identification of Signal Transduction Genes Responsible for High Altitude Stress

The different expression of genes involved in the signal cascade mechanism can affect the expression of genes participating in the formation of plant hormones such as auxin, ABA, ET, SA, and JA. Theses hormones may amplify the cascades or initiate some new signaling pathways (Sarwat et al., 2013; Wang et al., 2018). It is now known that these phytohormones extensively regulate all aspects of plant stress responses ranging from signal cascade transduction to modifications in plant developmental processes (Shu et al., 2018). In this experiment, GA, IAA, and JA were the top three responsive hormone signal transduction pathways in oats and there were a number of DEGs involved in them. Studies had shown that the gibberellin receptor sensing the GA signal could activate the signaling pathway, thereby regulating the expression of downstream genes to affect plant growth and morphogenesis (Ito et al., 2018). Totally 60 DEGs involved in the response to GA signal transduction pathway were identified as being differentially expressed, which encoded gibberellin receptor GID and DELLA proteins. This indicated that high altitude-induced expression of oats appeared to be GA dependent.

Auxin is a plant hormone extensively involved in regulating cell growth, cell division and cell-specific differentiations to participate in a series of growth and development processes (Tuan et al., 2019). ARF and SAUR family protein could bind to the cis-acting element of auxin-inducible gene and regulate its transcriptional expression (Spartz et al., 2014; Zhang et al., 2019). Forty-eight DEGs encoding three key proteins (transport inhibitor response, auxin-responsive and SAUR family protein) of IAA signaling pathway were identified in our study. It could infer that both ABF and SAUR can activate the down-stream gene expressions to respond to high altitude stresses.

JAs are a group of important hormones that play pivotal roles in a variety of plant growth, developmental and stress response processes (Sharma and Laxmi, 2015). In this study, 41 DEGs encoding jasmonate ZIM domain-containing protein and transcription factor MYC2 were enriched in the JA signaling pathway. It had been shown that JAZ gene and MYC2 were the major regulators of jasmonic acid signaling pathway in which JAZ protein degradation could release MYC2 transcription factor and activate the expression of downstream genes of jasmonic acid signaling pathway (Boter et al., 2004).

### High Altitude Resistant Genes Associated With Cutin, Suberine, and Wax Biosynthesis

The cuticle protects the surface of plant organs and plays a crucial role in the contact between plants and the environment. High altitude tolerant genes associated with cutin, suberine, and wax biosynthesis In many plants under salt stress, cuticle wax content is strongly negatively associated with cutin transpiration, suggesting that a large volume of cutin wax accumulation forms a protective barrier for controlling cutin transpiration, maximizing water usage quality, and improving the resistance of plant salt (Kosma et al., 2009; Hasanuzzaman et al., 2017). Plants can respond by increasing or decreasing the content of wax to high temperature stress. If the wax content of the cuticle is increased or decreased by high temperature stress is still inconclusive, which is related to the form of plant (Awika et al., 2017; Huggins et al., 2018). A significant number of cutin, suberine and wax biosynthesis genes were expressed differentially at elevated altitudes in Table 5. The results of the study on cuticle wax involved in the abiotic stress mechanism are clear, but there are few studies on high stress at altitude. Therefore, in reaction to high altitude stress, we investigated the molecular function of cuticle wax. The deposition of wax may be caused by stomatal growth to increase plant tolerance, suggesting that the improvements in wax synthesis are closely connected to irregular epidermal stomata. We found in our research that the density of stomata has changed significantly (Figure 1L). We therefore speculate that oats will change the density of stomata to regulate wax biosynthesis, further change the loss of stomata and non-stomata water, and ensure that the water content adjust to the condition of the plateau. This presents new ideas for the study under high altitude stress of the physiological and molecular processes of oats.

## Materials and Methods

### Plant Materials and Growth Conditions

Seeds of *Avena sativa* L. Qingyin No. 1 were collected from the Qinghai with the permission of the Qinghai Academy of Animal Science and Veterinary Medicine in the Qinghai Province of China. Animal Science and Veterinary Medicine in the Qinghai Province of China undertook the formal identification of the samples and provided details of specimen deposited. In order to investigate the effects of altitudes on the physiology and the transcriptions, oat leaf samples of the cultivar (cv.) “Qingyin No.1” were sown and collected at heading stage, 2015 in two regions with significant differences in altitude in Qinghai Province, China, County of Minhe, (36°14′, 102°41′) at the altitude ca. 2,080 m (defined as the low altitude) and County of Huangzhong (36°21′,101°44′) at ca. 2,918 m (defined as the high altitude). During the heading period, the climate data in the two altitude regions were recorded (Supplementary Table S11). Compared with low-altitude areas, high-altitude areas have longer sunshine duration and lower average monthly temperatures. The field plots were arranged as a completely randomized block design and adopted line sowing method. The distance of row spacing and seeding depth of were 20 and 5 cm, respectively. The experiment was carried out without fertilization and irrigation and weeding manually. The whole flag leaf was collected randomly and immediately frozen in liquid nitrogen. Samples were kept at −80°C for further analyses.

### The Measurements of Agronomic Traits

Plant heights were measured at heading. Each of the 5 oat plants in each plot was selected randomly to measure its height to calculate their mean. At the same stage, the ratio of stem to leaf was measured. About 0.5 kg of oat samples were weighed in each plot, and stems, leaves and inflorescences were separated into two parts. After air-dry, samples were weighed and the ratio of stem to leaf was calculated (repeated with above). The leaf sheath of the oat grasses was collected into stems and the ears into leaves. The fresh grass samples were naturally air-dried for the dry weights which were converted into hay yield per hectare. After weighed, the hay samples were crushed and sieved with 40-mesh then the crude fat (EE) was determined by the soxhlet extraction.

### Measurements of the Stomatal Density and Physiological Analyses

The stomatal density of oats grown at the two altitudes were observed by SEM (Bello et al., 2017).

SOD activity was detected by recording the decrease in optical density of NBT dye by the enzyme. MAD content was measured using a modified TBA method. H_2_O_2_ levels, O_2_^–^ activities, CAT, APX, GR, and Proline contents were tested using the related biological assay kits (Nanjing Jiancheng BioengineeringInstitute, China) following the manufacturer’s instructions with some modifications according to Tang et al. (2013). Approximate 0.1 g of each leaf sample was incubated on ice for 30 min and immersed in 10 ml of 95% ethanol in 25 ml brown flask at room temperature. Until the green color disappeared, the solution was set to volume, shaken, and stored in the dark. A series of 100 μl aliquots were taken every 10 min after initial immersion, and subjected to spectrophotometry (absorption measured at 647 and 664 nm) to quantify the amount of chlorophyll leached.

Before Fv/Fm measurement, whole oat plants were dark-adapted for 20 min, and Fv/Fm was measured using a steady-state gas-exchange system with an integrated fluorescence chamber head (Heinz Walz GmbH,Effeltrich,Germany). Fv/Fm values were calculated as Fv/Fm = (Fm – Fo)/Fm, NPQ = F m/F m ‘^–1^.

All assays described above were repeated four times, with four biological replicates. The data, which are shown as the means ± SDs, were subjected to ANOVA to determine significant differences. The least significant differences (LSDs) of the means were determined via Duncan’s test at the level of significance defined as α = 0.05.

### Library Preparation for Transcriptome Sequencing

RNA extraction was performed according to the manufacturer’s protocol of the RNA prep Pure Plant kit (KangWei Biotech Co., Ltd., Beijing, China). RNA purity and integrality were checked before the cDNA library construction. The mRNA was enriched from the total RNA through the magnetic beads with oligo (dT). Afterward, mRNA was randomly broken into fragments. Catalyzed by reverse transcriptase, double-stranded cDNA was synthesized through mRNA by combining with random primers. After ds-cDNA purified, the adapters and poly (A) tails were ligated at both ends of ds-cDNA. With the adapter sequences, ds-cDNA fragments were amplified through PCR. After ds-cDNA quantification, the cDNA libraries were ready for sequencing. using the Illumina HiSeq 2000 (single strands for sequencing).

### Transcriptome Assembly and Gene Function Annotations

The clean data were obtained by removing reads containing adapters and low quality ones from raw data. Transcriptome assemblies were performed using Trinity (Grabherr et al., 2011) based on clean data. The sequences of unigenes were compared with Nr (NCBI non-redundant), Nt (Nucleotide Sequence Database), SwissProt (a manually annotated and reviewed protein sequence database), and GO (Gene Ontology), KOG (euKaryotic Orthologous Groups) databases by BLAST and the KEGG (Kyoto Encyclopedia of Genes and Genomes). GO enrichment analysis was implemented by the Goseq R package on the basis of Wallenius non-central hypergeometric distribution. KOBAS software was subsequently used to test the statistical enrichment of DEGs in the KEGG pathways (Altschul, 1997).

### Differential Expression Analyses

The EdgeR program package was used to adjust read counts of each sequenced library and the RSEM package for differential expression level analyses of two samples. The *p*-value was adjusted using the *q*-value. In this study, |log2 (fold change)| > 2 were set as the thresholds for significantly differential expressions.

### Validation of RNA-Seq Data by qRT-PCR

The RNA samples isolated above were used as templates and were reverse transcribed with a HiScript II Q Select RT SuperMix for qPCR (gDNA eraser) kit (Vazyme, China). The expression of the actin gene was used as an internal control. qRT-PCR was performed with ChamQ^TM^ Universal SYBR qPCR Master Mix(Vazyme, China) on a LightCycler 480 II58 device (Roche, Switzerland) according to the manufacturers’ protocol. Relative gene expression levels were evaluated according to the 2^–ΔΔ*CT*^ method.

## Conclusion

In order to understand the mechanisms of oats adapting to high altitudes, we accomplished physiological and agronomical characterizations and transcriptome sequence analysis. The physiological and agronomical results showed that oats displayed significant increases in MDA, SOD and soluble sugar contents, accompanied by decreases in chlorophyll, NPQ, stomatal density, and hay yield. The transcriptomic profiles showed apparent differences between low and high altitudes where many pathways associated with high altitude stresses were identified including photosynthetic pigment synthesis, photosynthesis, and plant hormone signaling transduction pathways. Moreover, large amount of differentially expressed TFs between low and high altitudes was identified to participate in the response to altitude stress of oats, which can serve as candidate genes for altitude stresses molecular breeding project of oat. Through molecular technology of gene cloning or gene silencing to further clarify the functions of candidate genes and identify potential target genes that increase oat yield and photosynthesis under high altitude stress in oats. Association analysis of candidate gene can find marker sites that are significantly associated with agronomic traits of oats to improve breeding efficiency. In general, this results provided the basis for understanding better the biological regulation on oats under two scenarios of altitudes and thus contributed to breeding efforts aimed at increasing yield under such situations.

## Data Availability Statement

The raw sequencing data have been submitted to the NCBI Sequence Read Archive database with accession number PRJNA639320.

## Author Contributions

YJ, CG, and CY conceived and designed the experiments. YJ, LB, ST, HJ, KZ, and LL performed the experiments. HW, HT, HX, and LZ analyzed and interpreted the sequence data. YJ wrote the manuscript. All authors read and approved the final manuscript.

## Conflict of Interest

The authors declare that the research was conducted in the absence of any commercial or financial relationships that could be construed as a potential conflict of interest.

## Supplementary Material

Supplementary Table 1 Function annotation results of oat.

Supplementary Table 2 Annotation of 11,639 significantly differentially expressed genes transcripts.

Supplementary Table 3 Gene Ontology (GO) functional annotation of transcripts;

Supplementary Table 4 KEGG pathway mapping.

Supplementary Table 5 DEGs associated with TFs between low and high altitude.

Supplementary Table 6 Differently expressed genes related to porphyrin and chlorophyll metabolism.

Supplementary Table 7 Differently expressed genes related to carotenoid biosynthesis.

Supplementary Table 8 Differently expressed genes related to photosynthesis.

Supplementary Table 9 Differently expressed genes related to hormone signal transduction pathway.

Supplementary Table 10 20 differently expressed genes transcript abundance.

Supplementary Table 11 Meteorological data of the growing season of Minhe and Huangzhong County (April–October 2015).

## Abbreviations

NPQ: non-photochemical quenching coefficient
DEGs: differentially expressed genes
GO: Gene ontology
KEGG: Kyoto Encyclopedia of Genes and Genomes
Nr: NCBI non-redundant
Nt: Nucleotide Sequence Database
SwissProt: a manually annotated and reviewed protein sequence database
KOG: euKaryotic Orthologous Groups
TFs: transcription factors
Chl a: chlorophyll a
APX: ascorbate oxidase
CAT: catalase
GR: glutathione reductase
MDA: Malondialdehyde
SOD: superoxide dismutase
ROS: Reactive oxygen species
qRT-PCR: Quantificational real-time polymerase chain reaction
RNA-seq: RNA sequencing
SEM: scanning electron microscopy
IAA: Auxin
ABA: Abscisic acid
ET: Ethylene
SA: salicylicacid
JA: jasmonic acid
ARF: Auxin response factor
NBT: nitro-blue tetrazolium
TBA: thiobarbituric acid
FPKM: per Million mapped reads
RSEM: RNA sequencing by expectation maximization.
